# Consistent Strategy Updating in Spatial and Non-Spatial Behavioral Experiments Does Not Promote Cooperation in Social Networks

**DOI:** 10.1371/journal.pone.0047718

**Published:** 2012-11-19

**Authors:** Jelena Grujić, Torsten Röhl, Dirk Semmann, Manfred Milinski, Arne Traulsen

**Affiliations:** 1 Grupo Interdisciplinar de Sistemas Complejos (GISC), Departamento de Matemáticas, Universidad Carlos III de Madrid, Leganés, Madrid, Spain; 2 Evolutionary Theory Group, Max-Planck-Institute for Evolutionary Biology, Plön, Germany; 3 Research Group Evolution of Cooperation and Prosocial Behaviour, Courant Research Center Evolution of Social Behavior, Göttingen, Germany; 4 Department for Evolutionary Ecology, Max-Planck-Institute for Evolutionary Biology, Plön, Germany; University of Maribor, Slovenia

## Abstract

The presence of costly cooperation between otherwise selfish actors is not trivial. A prominent mechanism that promotes cooperation is spatial population structure. However, recent experiments with human subjects report substantially lower level of cooperation then predicted by theoretical models. We analyze the data of such an experiment in which a total of 400 players play a Prisoner's Dilemma on a 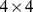 square lattice in two treatments, either interacting via a fixed square lattice (15 independent groups) or with a population structure changing after each interaction (10 independent groups). We analyze the statistics of individual decisions and infer in which way they can be matched with the typical models of evolutionary game theorists. We find no difference in the strategy updating between the two treatments. However, the strategy updates are distinct from the most popular models which lead to the promotion of cooperation as shown by computer simulations of the strategy updating. This suggests that the promotion of cooperation by population structure is not as straightforward in humans as often envisioned in theoretical models.

## Introduction

Why would a self-interested individual pay towards the welfare of someone else? The evolution of cooperation is a fascinating problem originating in evolutionary biology [1]–[3] which has extended to several other disciplines subsequently [4]–[7]. While the evolution of cooperation requires an explanation, several mechanisms have been proposed that are routinely invoked to explain it [8], [9]. One of them which is particularly popular among theorists is spatial population structure. Regular lattices lead to interesting effects and dependences on details of the underlying evolutionary model [10]–[20]. The exploration of non-regular population structures, such as scale-free networks, suggest an intricate dependence on details of the population structure and update mechanisms [18], [21]–[26]. Even more complex effects arise when the underlying population structure is dynamic [27]–[32].

The promotion of cooperation based on population structure has been analyzed extensively by an enormous number of mathematical and computational models. Many theoretical papers suggest a direct applicability to human behavior. But until now, only few experiments to test these predictions have been performed, as discussed in [33]. Such behavioral experiments have been performed on one-dimensional lattices [34], two dimensional lattices [35]–[37], and complex networks [37]–[39]. These studies have tested the predictions of theoretical models, i.e. the level of cooperation induced by population structure, and also the underlying assumption of update mechanisms.

So far, there is little evidence that the sophisticated theoretical results of cooperation in structured populations can be carried over directly to human behavior. One important question from the perspective of a theoretician is whether human subjects condition their decision making on the population structure, i.e. whether they use consistent strategy updating in spatial and non-spatial experiments. Here, we analyze the data of such a behavioral experiment with a two-dimensional lattice and fully independent controls to address this issue [35]. Previously, this data has only been used to infer the strategy updating in the spatial system, but no systematic comparison between the treatments has been provided. We find no significant difference between the spatial and the non-spatial treatment in strategy updating, which suggests that the subjects did not adapt their behavior to the population structure. However, the way that strategies are updated is different from the update rules usually used in theoretical models that promote the evolution of cooperation.

## Results

### Experimental setup

The classical Prisoner's Dilemma is played between two players, each of them can choose to cooperate (C) or to defect (D). The payoff matrix is given by
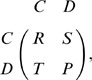
(1)where the shown payoffs are for the row player. Here, 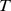 stands for the temptation to defect, 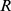 for the reward for mutual cooperation, 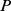 for the punishment for mutual defection and 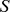 for the sucker's payoff. A Prisoner's Dilemma is defined by the inequation 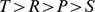. In other words, while mutual cooperation leads to a higher payoff than mutual defection, it is worthwhile to defect against a cooperator (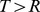) and to defect against a defector (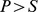), In addition to this payoff ranking, the condition 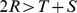 should be added in repeated games. In the experiment we have analyzed, the payoffs were chosen as 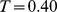 €, 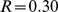 €, 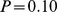 € and 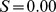 €.

In the vast majority of spatially extended models, players make only a single decision in each round which determines their action against all their neighbors. The same holds for our experiment. Participants of the spatial treatment discussed here were virtually located on the nodes of a 4
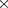
4 square lattice with periodic boundary conditions, as if the players would be located on the torus. They play a PD game with each of the four neighbors in their von Neumann neighborhood (the four cells orthogonally surrounding a central cell on the lattice). Players must choose one strategy which determines their action in all four games with their four neighbors. The payoffs are calculated by adding the four payoffs of individual games with each neighbor. There are no self-interactions. After each round, players were informed about their action and payoff as well as the actions and payoffs of their four neighbors. Based on this information and their experience from previous interactions, they had to decide on their next action.

The experiment had 25 sessions separated in two different treatments. In the spatial treatment the players had fixed neighbors, which stayed the same throughout the whole game. This treatment was repeated 15 times, each with 16 players and 25 rounds. In the non spatial control treatment (repeated 10 times with 16 players and 25 rounds), the neighbors were assigned to a new, random location on the lattice after each round and consequently, the neighbors of each player changed in each round. Players were not informed about the number of rounds. In both treatments, players are informed about the outcomes of every round, about the actions and payoffs of the neighbors they played with. However, at the moment they need to make a decision about their next action, they are not informed about the previous actions or payoff of their new neighbors. In contrast it was easy to remember the previous actions of the neighbors in the spatial setting.

### General observables in the two treatments

Let us compare the general outcomes of the spatial and non-spatial treatments.

**Figure 1:**
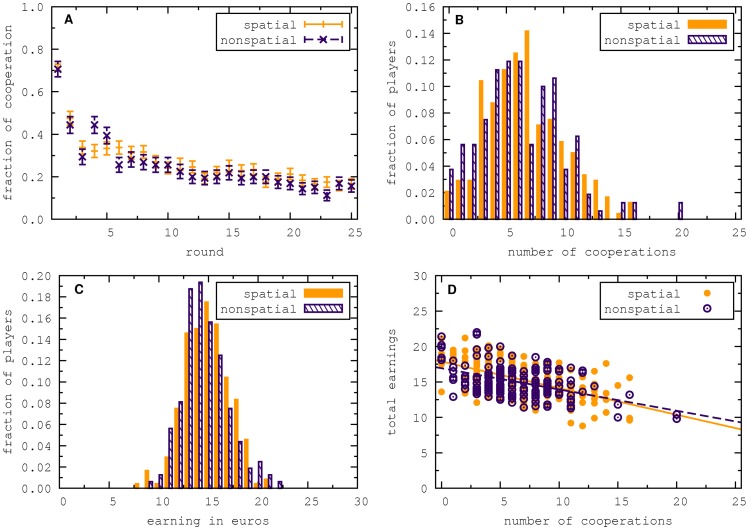
Comparison of the spatial and non-spatial treatments. (a) The fraction of players that have chosen to cooperate is decreasing over time, but remains substantial throughout the experiment [35]. The error bars are the standard deviations of a binomial distribution, 
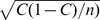
, where 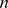 is the number of samples and 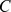 is the fraction of cooperation. (b) The distribution of cooperative acts per player. We do not observe unconditional cooperation, and very little unconditional defection (5 out of 240 players in the spatial treatment and 6 out of 160 players in the non spatial treatment). (c) The distributions of cumulative payoffs are peaked with median of 15.4 € for the spatial and 15.0 € for the non spatial treatment. The standard deviation is 2.3 € in both cases. (d) Correlation between the frequency of cooperation on the x-axis and the cumulative payoff on the y-axis. Each point is one player.

We find no significant differences in the fraction of cooperative actions between the two treatments. Figure 1 illustrates that the errors bars of the treatments are overlapping to the great extend, which suggests that there are no large differences between the treatments. This can be backed up by several statistical tests. First, we fit the difference between the two treatments with a linear function. We find an intercept of 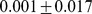 and a slope 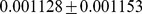. Since both values are smaller than their errors, it suggests that the values are close to zero. Second, we constructed a nonlinear regression model with a dummy variable for the spatial and the non spatial treatments. In this model, the fraction of cooperative actions 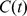 in round 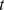 is given by
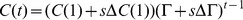
(2)Here, the parameters of the model are 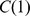, measuring the fraction of cooperative actions in the first round of the non spatial treatment, 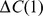, measuring the difference in cooperative actions in the first round between the two treatments, 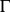, measuring the decay on cooperative actions in the non spatial treatment, and 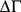, measuring the difference in this decay between the two treatments. In addition, we introduced the dummy variable 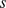, which equals 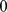 for the non spatial treatment and 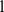 for the spatial treatment. From the nonlinear regression model, we find 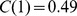 (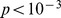) and 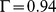 (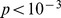). The 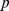-values show that these numbers are significantly different from zero. For the differences, we obtain 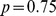 for 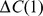 and 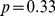 for 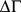, showing that the dependence on the dummy variable is not statistically significant. All this indicates that the difference between two treatments is not significant.

Next, we address the distribution of cooperative acts per player, the distribution of cumulative payoff per player, and the correlation between the two. Figure 1 illustrates that these two distributions are very similar in the two treatments. To compare the distributions between the treatments quantitatively, we performed a Kolmogorov-Smirnov test. We found 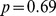 for the comparison of the two distributions of cooperative acts and 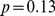 for the comparison of the two distributions of cumulative payoffs. These 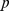-values indicate that we cannot accept the hypothesis that the two distributions arising from the two treatments are different. In order to compare the correlation between the cumulative payoffs and the number of cooperative acts, we developed a linear regression model,
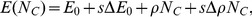
(3)where 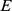 is the cumulative payoff, 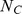 is the number of cooperative acts, 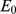 is the intercept for the non spatial treatment and 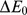 the difference between the intercepts of the two treatments. The slope in the non spatial treatment is measured by 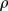 and 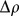 measures the difference of the slope between the two treatments. Again, 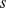 is a dummy variable which is equal to 0 for the non spatial treatment and 1 for the spatial treatment. We obtained 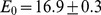 (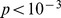), 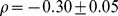 (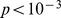), 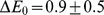 (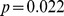), and 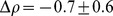 (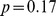). The large 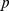 values for 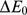 and 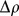 show that there is no significant difference between the two treatments.

In the next section, we depart from the level of aggregate information on the system level and address the individual decisions of our players in more detail. Many theoretical models have shown that this kind of update mechanism can have a profound impact on the outcome in such models [16], [17], [23], [26].

### Update mechanisms

In order to understand the dynamics of the system in more detail, we fitted three different update mechanisms that are popular in theoretical studies to the data of the experiment

- Unconditional imitation, where each players switches to the strategy that performed best in the past in the neighborhood. In addition, we assume that some decisions are made at random and that this fraction changes over time.
- Fermi rule - where strategies with higher payoffs are imitated with higher probability. In addition, sometimes a random strategy is chosen.
- Moody conditional cooperation - Cooperation conditioned upon the own action in the previous round and the number of cooperators in the neighborhood.

**Figure 2:**
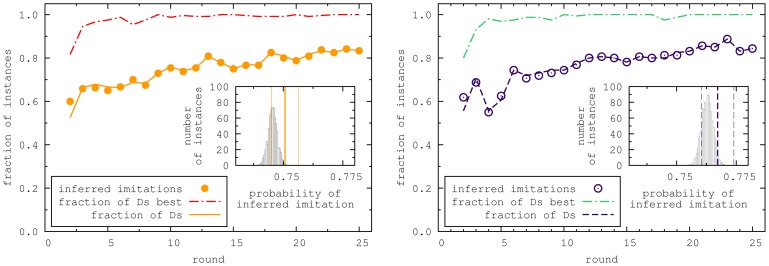
Unconditional imitation test in the spatial treatment (left) and in the non spatial treatment (right). The main panels show three different type of data: the fraction of inferred imitations, the level of defection and fraction of decisions in which defection was the best performing strategy in the neighborhood. The inferred level of imitation is the fraction of actions in which the players action coincided with the action of the best neighbor in the previous round. Since defection is almost always the best performing strategy, a defecting player seems to be imitating. Therefore, the level of defection is almost identical to the level of the inferred imitations. However, the randomization test illustrated in the inset shows that there is still more imitation than expected in a random setting. The vertical lines show the inferred imitation observed in the experiments, plus and minus a standard deviation, and the gray bars show the distribution of the inferred imitation in the randomized sample.

In the classical studies on the promotion of cooperation on lattices, unconditional imitation has been assumed [10], [12], [40]. In this case, players update their strategies by imitating the previous action of the neighbor with the highest payoff. In Figure 2, we illustrate how often the players action is the same as the action of the highest scoring neighbor in the previous round. The probability of this inferred imitation is around 75% and is growing during the game. However, before we conclude that the unconditional imitation is the update mechanism players use frequently, we should notice that defection is almost always the most successful strategy in the neighborhood. Therefore, if a player defects it seems that she/he is imitating the best neighbor. Consequently, the level of defection is very similar to the level of inferred imitation (Figure 2). To further test the hypotheses of unconditional imitation we performed a randomization test [41]. In this test, the action of the players is kept, but the neighborhood is randomized. This gives a reference model for imitation, because with randomized neighborhoods there can be no imitation. We repeated the randomization 10 000 times to compute the distribution of probabilities of inferred imitation from a random setting. The results are presented in the insets of Figure 2. We see that distributions are very narrow and that the value from the experiment is slightly higher than the randomized average. The p-value is 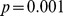 for the spatial treatment and 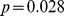 for the non spatial treatment, indicating that the small difference between the observed imitation and the randomized one is significant. This is different from the result of the same analysis in [36], where the small difference is not significant. However there are several differences between the two experiment which could be responsible for this disagreement, such as sample size, system size, number of neighbors, payoff matrix etc. Importantly, in both experiment the level of imitation was lower then 80%. Thus, players sometimes use strategies not played in their neighborhood before, which is very likely to prevent the cluster formation in both cases equally.

**Figure 3:**
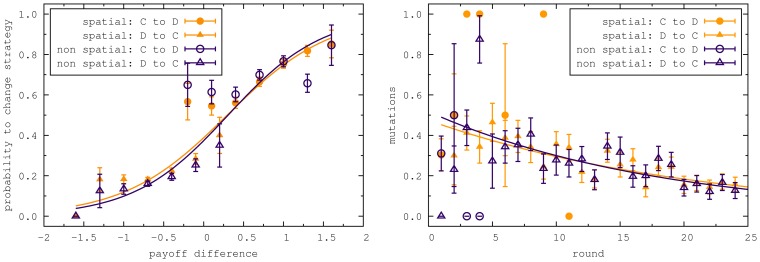
Probability of imitating depending on the payoff difference. Left: probability of switching to another strategy depending on the payoff differences for both the spatial and non spatial experiment. The payoff difference is between the focal player and the best player of the opposite strategy. The results are consistent with imitating the neighbors with higher payoffs. However this imitation is not unconditional, but the higher the payoff difference the larger is the probability of imitation. In addition, players might spontaneously switch their strategies even if they have no neighbors playing the other strategy, resembling mutations. Error bars are the standard deviations of a binomial distribution, 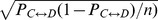, where 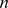 is the number of samples and 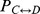 is the probability of changing the action). Right: Probability of mutations in time. Mutations are defined as the probability that a cooperator surrounded by four cooperators would change the strategy in the next round or that a defector surrounded by four defectors will change the strategy in the next round. We see a large number of mutations, which decreases over time, but always stay substantial. Again in both treatments the players show a similar pattern of behavior. Error bars are the standard deviations of a binomial distribution, 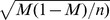, where 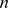 is the number of samples and 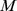 is the probability of mutation).

The second mechanism we tested is typically referred to as Fermi rule [11], [14], [42]. For this rule, the better the neighbor performs the higher is the possibility that he/she will be imitated, see Fig. 3. Here, 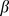 measures the intensity of selection, for 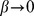 imitation is random and for 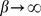, we recover the unconditional imitation from above. Note that this is slightly different from the original Fermi update mechanism, which would be difficult to check. In the original mechanism a random player is chosen and then imitated with the probability given above. However, the additional randomness would make it difficult to analyze the original rule in the experimental data, because in two identical situations, two players could chose a different payoff difference as the basis for comparison. Therefore we measure the probability of imitating the most successful neighbor who played the opposite strategy instead. This test corresponds to a rule where instead of the random player, the best player of the opposite action is chosen and imitated with the same probability as in the original rule. However the rule conserves a very important property of the original rule, which is to allow strategy changes even when the payoff difference is negative, since the best performing player of the opposite strategy can still have a payoff smaller than the focal player. To analyze this dependence, we again fitted the data to a logistic regression model,
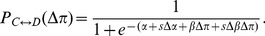
(4)Here, 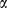 measures the probability to switch strategy in the case of zero payoff differences and 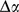 measures the difference between this quantity in the two treatments. The parameter 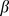 measures the intensity of selection and 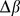 is the difference in the intensity of selection between the treatments. As above, 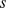 is a dummy variable with 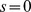 for the non spatial and 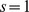 for the spatial treatment. The 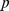-values for 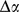 and 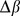 are 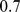 and 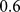, respectively, such that the dependence on the treatment is not significant.

However, the players will switch their strategies even if they are surrounded by players with the same strategy as theirs. This corresponds to mutations or exploration behavior [43]. This exploration behavior decreases over time in a manner comparable with the decrease of global cooperation level (Figure 3). To analyze the difference between the spatial and non spatial treatment, we utilize a non linear regression model,
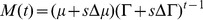
(5)where, 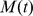 is the fraction of exploration behavior in round 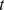. The initial level of exploration is measured by 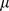 and 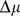, its decay is measured by 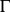 and 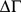. The p-values for the parameters 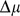 and 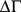 are both 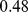. Thus, the dependence on the treatment is statistically not significant.

**Figure 4:**
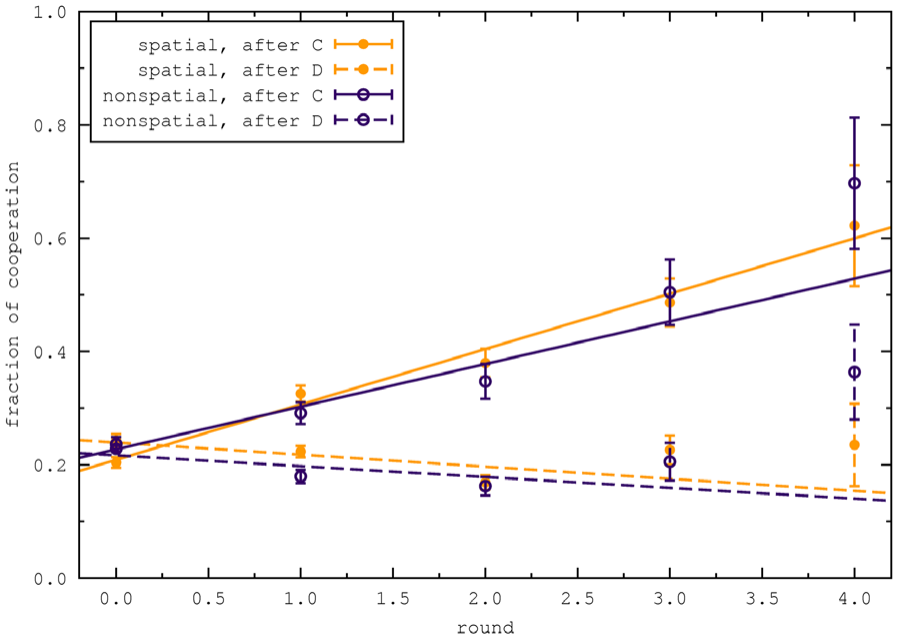
Probability of cooperation depending on the previous action and the number of cooperators in the neighborhood. These are called moody conditional cooperators in [36]. We see that there is a clear difference between the behavior after cooperating and defecting. After cooperating, the probability of cooperating increases with the number of cooperating neighbors and after defecting the probability of cooperation is decreasing with the number of cooperating neighbors. Again, in both the spatial and the non spatial setting the behavior is very similar.

The last update mechanism we analyzed is conditional behavior based on the own previous action and the number of cooperators in the neighborhood. In [36], this has been termed “moody conditional cooperation”. In Figure 4, we show the probability of cooperating depending on the number of neighbors who cooperated in the previous round and the action of the focal players in the previous round. In the case that the focal player cooperated in the previous round, the probability of her/him cooperating increases linearly with the number of cooperating neighbors, as in previous work. On the other hand, if the player defected in the previous round, the probability of him/her cooperating decreases linearly with the number of cooperating neighbors. We developed a linear regression model with two dummy variables,
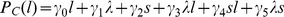
(6)where 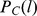 is probability of cooperation after 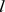 of your neighbors cooperated in the previous round. The 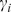 are the parameters of the model, in a similar manner as described above. Again, 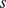 is a dummy variable which is equal to 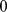 for the non spatial treatment and 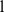 for the spatial one. The second dummy variable 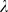 is equal to 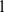 if the focal player cooperated himself/herself in the previous round and 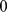 otherwise. 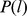 depends significantly only on 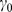 and 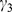 (p-values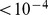). Therefore, the probability of cooperation does not depend on the treatment. It does not depend either on the number of cooperators in the previous round if we do not control for the players own action.

### Simulations

In the experiments, there is no hint for a significant difference between the treatments. In order to understand why this happens, we have performed simulations with the three update mechanisms fitted to the experimental data: unconditional imitation (with random strategy exploration), Fermi rule (also with random strategy exploration) and moody conditional cooperation. We found that these three update mechanisms will not promote cooperation on lattices and that for them spatial structure does not make a difference, even for much larger systems. Unconditional imitation with random strategy exploration obeys the equation
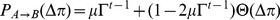
(7)where 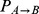 is the probability that a player with action A will change his action to the action of the player B, who is her/his best performing neighbor. The round of the game is 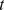, 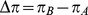, where 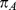 is the payoff of an 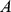 player and 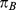 is the payoff of his best performing neighbor playing 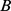. 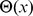 is the Heaviside function, which is one for positive arguments and zero otherwise. From the experimental data, we found 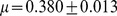 and 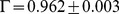. For the simulations with imitation only we set the random strategy exploration parameter 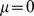. In the first round, 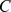 is played with probability 70% and in the every other round the probability of imitation the best player is determined according to the probability given by Eq.7.

If the player does not imitate she/he will play C or D with equal probability. We see that the simulations with 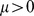 reproduce the cooperation level well, but as we saw before, the best performing neighbor will almost always be a defector. Therefore the above update mechanism is equivalent to the mechanism where the next action is determined only by the term 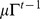 in Eq. 7. Promotion of cooperation can only occur through the formation of clusters of cooperators, which is prevented by the random strategy exploration. Therefore, since clusters of cooperators cannot be formed anyway, both spatial and non spatial treatments show low levels of cooperation driven by 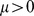 only. On the other hand, for 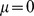, the two simulation setups display very different dynamics. In the spatial setting, the level of cooperation drops at the beginning, until clusters start forming and expand in a sufficiently large system. In the non spatial setting, such clusters cannot form and the cooperation level drops to zero.

In the Fermi update rule, the probability of switching to the opposite strategy depends on the difference of the payoffs between the focal player its neighbors. The dependence is given by the Fermi function, see above. While conventionally a random neighbor is chosen for comparison, in the analysis of the experimental data we have focused on the neighbor with the opposite strategy and the highest payoff. In our simulations, we take the same approach. If there are no players with the opposite strategy in the neighborhood, players will still switch their strategy with some probability. We call this mutations or exploration behavior. In contrast to [35], we here assume that this quantity is time dependent. In the right panel of Figure 3, we present the probability of mutations over time. Summarizing this approach we find for the probability of changing strategy
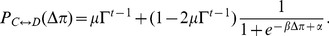
(8)Note that for 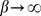, we recover the unconditional imitation from above. For the simulations, we used the parameters obtained from fitting to the experiment, 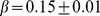, 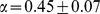, 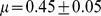, 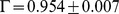 for the spatial treatment and 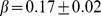, 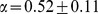, 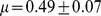, 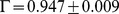 for the non spatial treatment.

The last model we simulated are the moody conditional cooperators. The probability of cooperating is given by
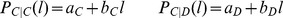
(9)The probability of cooperating after 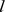 neighbors cooperated and the focal player cooperated is 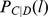. If the focal player defected, the corresponding probability is 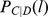. For the simulations, we chose the parameters 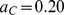, 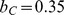, 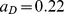, and 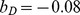.

**Figure 5:**
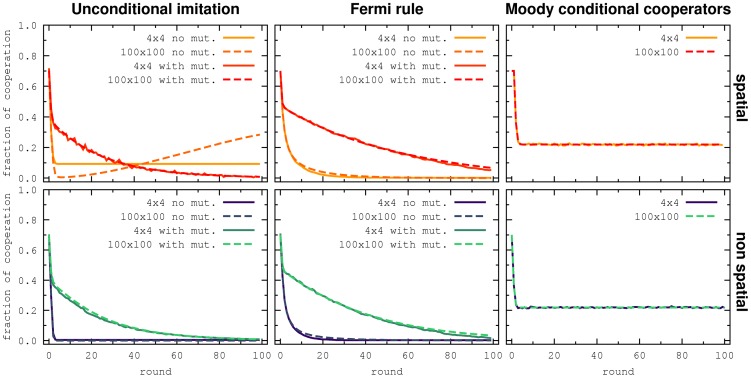
Simulations for different update mechanisms. Top figures are for the spatial structure and the bottom ones are for non spatial structure. Left to right: unconditional imitation, Fermi and moody conditional cooperators. We see that, for this payoff matrix, the only update mechanism where the spatial structure is relevant is unconditional imitations without random strategy exploration.

Simulations were performed for a spatial and a non spatial setting. In order to analyze the influence of the size of the lattice, we simulated lattice size 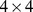 and 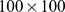. Figure 5 shows the levels on cooperation in these simulations for the same payoff matrix as in the experiments, and for the parameters obtained from the fit to the experimental data. As previously well established [10], unconditional imitation without exploration will promote cooperation in the presence of spatial population structure, but not in non spatial settings. But the promotion of cooperation will take place only if there is essentially no random strategy exploration in the system. The presence of noise in the strategy updating will also destroy the clusters of cooperators. Thus, the level of cooperation will be equal to the strategy exploration both in the spatial and the non spatial setting. On the other hand, the Fermi rule and the moody conditional cooperators rule lead to the same levels of cooperation in the spatial and in the non spatial setting for our choice of parameters. Notice, that this may change for a different payoff matrix, other parameters or if we would allow self-interactions. Our results are in good agreement with the results in [44], where it is shown by simulations that in a population of cooperators, defectors and moody conditional cooperators, the structure of the population does not promote or inhibit cooperation compared to a well mixed population.

## Discussion

We have compared a spatial and a non spatial behavioral experiment with human subjects playing a Prisoner's Dilemma. We have found no significant differences between the two treatments, neither in macroscopic properties such as the level of cooperation, nor in the way that players update their strategies. On the one hand, this is good news for theorists, because their assumption of consistent strategy updates in spatial and non spatial systems seems to be justified. On the other hand, our results suggest that the idea that spatial structure promotes cooperation cannot be carried over to human experiments in a straightforward way. This result is in line with previous results from other experiments. Cassar has found that cooperation was hard to reach on different, albeit small networks [38]. Kirchkamp and Nagel have performed an experiment on a one dimensional lattice (circles) as well as in a group setting; their results suggest that naive imitation may be negligible in such experiments [34]. Suri and Watts have performed an online experiment and found that network topology had no significant effect on the level of cooperation [39].

It could be argued that in the above experiments the cooperation is not observed because of the small system size, but in experiments one order of magnitude larger [36] or even two orders of magnitude larger [37], the level of cooperation changed over time similarly. However, in those experiments, the same players were used for both treatments subsequently. Therefore, the comparison we make here is not straightforward in those experiments. All these experiments are performed on different spatial structures with different payoff matrices and system sizes. Common to them is the observation that a strategy updating that does not allow the innovation of a new strategy in a neighborhood, e.g. players switching to defection in a neighbor of cooperators, is not a good explanation for the data.

It could be argued that the size of our system is not big enough for spatial structure to make a difference or that the payoff matrix is not the ideal choice. In principle, it is possible that for a different payoff matrix or a larger system, significant differences between the two treatments (spatial and non spatial) would be observed. Therefore, the similarity of players' behavior in both settings should be put to the test in larger systems and for different payoff matrices, before a final conclusion is made. However, one has to keep in mind that random strategy exploration is preventing the formation of clusters. It seems to be unlikely that this feature disappears for larger lattices or different parameters.

However, our results do not imply that the theoretical analysis of spatial games is not meaningful. In other biological or technological systems these considerations may be applicable directly. Moreover, the effect of spatial structure could be much more subtle than implied by many theoretical works. In particular, theoretical work should consider the role of mutations (which may arise from mixed strategies, strategies that try to anticipate the future behavior of the neighbors, or from strategies which consider more than one past interaction), see e.g. [43], [45], [46], or other, more sophisticated strategy update mechanisms [47]–[49], which is only rarely done in structured populations. However, it is difficult to imagine strictly spatially structured hunter gatherer society.

In addition, population structure may have played a crucial role in our evolutionary past and potentially also in our present and future [50]. It is frequently argued that many features of human behavior have evolved in hunter gatherer societies. The population structure of these societies may thus be crucial for the evolution of human cooperation.

Most importantly, theoretical considerations of fixed networks are a necessary first step to analyze dynamical networks, which may be a more realistic way to address human behavior. Recent experiments of such dynamical networks indicate that there is indeed a scope for the evolution of cooperation mediated by network structure [51], [52].

## Methods

We used the experimental data from [35]. A detailed explanation of the experiment can be found there. The data of this experiment is available upon request. We emphasize that each player was identified by a letter ranging from a to p (e.g., a has the following neighbors: b, d, e, and m). Therefore, in the spatial treatment players could see that their neighbors were always the same, for example: f, d, e, and a. Subjects were told in the instructions that their neighbors would stay the same throughout. On the other hand, in non spatial treatment, players could see that in each round they have different neighbors. Subjects were told in the instructions that their neighbors would change after each round. Consequently, it is highly unlikely that the players misunderstood their specific rules of the game. In the non spatial treatment the players had no access to information of the previous actions of their present neighbors. Thus, they could not react directly to their previous behavior. In contrast it was very easy in the spatial setting to memorize the four strategies of their neighbors for the next encounter.
